# Nutritional Status Evaluation in Patients Affected by Bethlem Myopathy and Ullrich Congenital Muscular Dystrophy

**DOI:** 10.3389/fnagi.2014.00315

**Published:** 2014-11-17

**Authors:** Silvia Toni, Riccardo Morandi, Marcello Busacchi, Lucia Tardini, Luciano Merlini, Nino Carlo Battistini, Massimo Pellegrini

**Affiliations:** ^1^Laboratory of Nutrition and Lifestyle, Department of Diagnostic, Clinical and Public Health Medicine, Modena, Italy; ^2^Laboratory of Musculoskeletal Cell Biology, Istituto Ortopedico Rizzoli, Bologna, Italy

**Keywords:** collagen VI, muscular dystrophies, nutritional assessment, body composition, basal energy expenditure

## Abstract

Collagen VI mutations lead to disabling myopathies like Bethlem myopathy (BM) and Ullrich congenital muscular dystrophy (UCMD). We have investigated the nutritional and metabolic status of one UCMD and seven BM patients (five female, three male, mean age 31 ± 9 years) in order to find a potential metabolic target for nutritional intervention. For this study, we used standard anthropometric tools, such as BMI evaluation and body circumference measurements. All results were compared to dual-energy X-ray absorptiometry (DXA), considered the “gold standard” method. Energy intake of each patient was evaluated through longitudinal methods (7-day food diary) while resting energy expenditure (REE) was predicted using specific equations and measured by indirect calorimetry. Clinical evaluation included general and nutritional blood and urine laboratory analyses and quantitative muscle strength measurement by hand-held dynamometry. BM and UCMD patients showed an altered body composition, characterized by low free fat mass (FFM) and high fat mass (FM), allowing us to classify them as sarcopenic, and all but one as sarcopenic-obese. Another main result was the negative correlation between REE/FFM ratio (basal energy expenditure per kilograms of fat-free mass) and the severity of the disease, as defined by the muscle megascore (correlation coefficient −0.955, *P*-value <0.001). We postulate that the increase of the REE/FFM ratio in relation to the severity of the disease may be due to an altered and pathophysiological loss of energetic efficiency at the expense of skeletal muscle. We show that a specific metabolic disequilibrium is related to the severity of the disease, which may represent a target for a nutritional intervention in these patients.

## Introduction

Mutations in the genes COL6A1, COL6A2, and COL6A3, coding for three α chains of collagen type VI, cause COL6-related myopathies (COL6-RM), including the severe Ullrich congenital muscular dystrophy (UCMD), the milder Bethlem myopathy (BM) (Bertini and Pepe, 2002; Allamand et al., 2010), and the Myosclerosis Myopathy in a single family (Merlini et al., 2008a,b).

The prevalence of UCMD and BM has been calculated as 0.13 per 100,000 and 0.77 per 100,000, respectively (Norwood et al., 2009). BM (MIM #158810) (Merlini et al., 1994) is characterized by axial and proximal muscle wasting and weakness with finger flexion contractures. BM is usually mild, sometimes slowly progressive (Pepe et al., 2002). BM has both dominant and recessive inheritance (Gualandi et al., 2009). Immunohistochemistry shows normal or mildly reduced levels of ColVI in the endomysium of most BM patients (Allamand et al., 2010). UCMD (MIM #254090) (Mercuri et al., 2005) is a severe congenital muscular dystrophy, characterized by early onset, generalized and rapidly progressive muscle wasting and weakness, proximal joint contractures, and distal joint hypermobility. Walking ability is rarely achieved or preserved during adolescence, and the rapid progression of the clinical symptoms usually leads to early death, due to respiratory failure (Mercuri et al., 2005). UCMD is caused both by recessive and *de novo* dominant mutations (Mercuri et al., 2005). ColVI appears to be strongly reduced or absent in muscle biopsies from UCMD patients.

A dystrophic mouse model, where collagen VI synthesis was prevented by targeted inactivation of the Col6a1 gene, allowed the investigation of the pathogenesis, revealing the existence of a Ca(2+)-mediated dysfunction of the mitochondria and sarcoplasmic reticulum and defective autophagy (Bernardi and Bonaldo, 2013). Similar defects contribute to the disease pathogenesis in patients, irrespective of the genetic lesion causing the collagen VI defect (Irwin et al., 2003; Grumati et al., 2010). These studies indicate that permeability transition pore opening and defective autophagy represent key elements of skeletal muscle fiber death, and provide a rationale for the use of cyclosporin A (Merlini et al., 2008a,b) and of nutritional interventions to correct defective autophagy (Merlini et al., 2014) in patients affected by COL6-RM, a strategy that holds great promise for treatment.

In the last decade, several studies have demonstrated that nutritional status and body composition are strictly related to clinical outcomes and that nutritional intervention can be effective in the prevention and treatment of many diseases related to metabolism, bioenergetics, and even cancer.

According to a three-compartment model of body composition (Moon et al., 2008), total body weight is the sum of lean mass (LM), fat mass (FM), and bone mineral content (BMC). The LM, which includes the mass of the internal organs and that of the muscles, together with the BMC, form the fat-free mass (FFM), which represents the more metabolically active component of the human body. A “pathological” ratio between FFM and FM and/or an abnormal distribution of these components in the body, mainly trunk versus appendages, is found in many pathological conditions and correlates with the severity of the metabolic or energy status alteration; moreover, modifications of body composition *per se* may represent an independent health risk factor (Wohlfahrt et al., 2014). Augmented FM leads to a higher cardiometabolic risk and to a higher incidence of hypertension, diabetes (Rohan et al., 2013), and cardiovascular diseases.

Muscular dystrophies (MD) are characterized by progressive deterioration of muscle mass, muscle strength, and function. Resting energy expenditure (REE), which comprises 70% of daily energy needs, is determined by the amount and composition of the metabolically active fat-free mass (FFM). The reduced muscle mass and muscular activity, characteristic of MDs, could result in a significant parallel decrease in REE. Surprisingly, it was found that that patients with Emery-Dreifuss MD (Vaisman et al., 2004) and Duchenne and Becker MD (Zanardi et al., 2003a; Gonzalez-Bermejo et al., 2005; Hogan, 2008; Elliott et al., 2012) may have increased energy expenditure. If not met with increased caloric intake, this greater energy expenditure may partially contribute to further deterioration in their muscle mass and function.

It has already been shown that patients with COL6-RM have reduced muscle mass, muscle strength, and muscle function (Miscione et al., 2013). To date, no data are available on the energy expenditure in COL6-RM. The aim of this study is to investigate the relationship between REE, body composition, and muscle strength in COL6-RM.

## Materials and Methods

### Patients

We analyzed eight adult patients (five women, three men, mean age 31 ± 9 years) with COL6-RM: seven had BM and one UCMD. The UCMD patient was never able to walk and was on nocturnal non-invasive mechanical ventilation. This study was approved by the institutional ethical committee of the Istituto Ortopedico Rizzoli (ClinicalTrials.gov identifier: NCT01438788). All subjects were fully informed about the study and gave their written informed consent.

### Body composition

Nutritional status was evaluated throughout the study by both non-invasive and invasive techniques (El Ghoch et al., 2014). Body composition was obtained by DXA (Hologic 4500 W; software version 11.2; Hologic, Inc., Waltham, MA, USA) software, which provides regional and whole-body estimation of LM, FM, and BMC, according to the three-compartment model of body composition. FFM was calculated as the sum of LM and BMC, and has been provided for each body part (i.e., trunk and limbs) (Scaglioni et al., 2013). From these whole-body measures, the following derivative values were calculated: FMI (FM/height2), LM/height2, appendicular lean mass/height2 (ALMI). Appendicular lean mass (ALM) was the sum of bone-free and fat-free tissue masses in the arms and legs. Sarcopenic obesity was defined, according to Baumgartner et al. (2004), as ALM divided by stature squared (ALMI) less than 7.26 kg/m^2^ in men and 5.45 kg/m^2^ in women and percentage body fat, derived by DXA, greater than 28% in men and 40% in women (Baumgartner et al., 2004).

Anthropometric measurements included: body weight (Wt), height (Ht), and circumferences [waist, hip, waist-hip ratio (WHR)]. All measurements were determined by the same operator following the Anthropometric Standardization Reference Manual recommendations (Lohman et al., 1988). Body mass index (BMI) was calculated as Wt [Kg/Ht(m^2^)]. We used BMI to categorized participants as obese (BMI ≥30), overweight (25 ≤ BMI < 30), normal weight (18.5 < BMI < 25), or underweight ≤18.5.

### Energy and nitrogen balance

Resting energy expenditure was estimated by indirect calorimetry using a metabolic measurement cart with a canopy hood (CareFusion Vmax Encore, San Diego, CA, USA). Subjects were instructed to fast for 12 h and abstain from exercise for 24 h before the test (Mifflin et al., 1990). Before measuring REE, all subjects were asked to rest quietly in the supine position for approximately 30–40 min in an isolated room, with a temperature between 21° and 24°C. The criterion for a valid REE was 15 min of steady state, determined as <5% variation in respiratory quotient (RQ)/minute and oxygen consumption/minute. Oxygen consumption and carbon dioxide production were used to calculate REE, in accordance with the Weir equation (Turell and Alexander, 1964). REE were also calculated with the equations based on weight, height, age, and sex [Harris-Benedict and Schofield (Energy and protein requirements. Report of a joint FAO/WHO/UNU expert consultation, 1985; Roza and Shizgal, 1984)] using the free fat mass (FFM)-based predictive equations of Mifflin and Katch and McArdle (McArdle et al., 1986; Mifflin et al., 1990).

Food intake was evaluated by a 7-day food diary and a dietitian interview (O’Connor et al., 2014). Nitrogen balance, an important tool for estimating adequate protein intake (Tarnopolsky et al., 1988), was calculated as the difference between nitrogen input (24 h dietary protein intake) and nitrogen output (24 h urinary urea nitrogen).

General and nutritional blood and urine laboratory analyses (albumin: transferrin; creatinine; uric acid; glucose; triglycerides: total, HDL, and LDL cholesterol; urinary creatinine; and nitrogen) were taken to assess the metabolic status of the patients. The 24-h urinary creatinine excretion value was used as an index of protein nutrition; the creatinine height index (CHI) and lean body mass was estimated from this value.

### Muscle strength

A composite score (megascore) was calculated by summing the maximal force of eight physical tests (Bryan et al., 2003; Merlini et al., 2003) using a hand-held dynamometer (Type CT 3001, Citec, C.I.T. Technics BV, Groningen, The Netherlands) (Van der Ploeg et al., 1991; Beenakker et al., 2001). Four muscle groups were examined bilaterally: hand grip, elbow flexors, knee extensors, and knee flexors (Merlini et al., 2002, 2003, 2004). Each individual muscle group was tested for at least 3 s using a “make” test (Merlini et al., 2004). The maximum force from three attempts was used in the analysis.

### Statistic analysis

Pearson correlation coefficients were calculated to study the association between different parameters. Statistical significance was set at 0.05. All analyses were conducted using the STATA software package for Windows 13.1 (Stata Corp, College Station, TX, USA). Measurable variables are presented as mean ± SD and categorical data as number and percentage.

## Results

### Anthropometric evaluation

Anthropometric analysis showed that UCMD and BM patients have average BMI values in the range of normality, comparable with a healthy population (Janssen et al., 2002). Body circumference measurements do not show any significant variations from normality (Janssen et al., 2002) (Table 1).

**Table 1:** **Anthropometric analysis**.

	M (*n* = 3)	W (*n* = 5)
Body weight (Kg)	67.40	61.24
	(±11.39)	(±12.34)
Body height (m)	1.72	1.63
	(±0.01)	(±0.05)
BMI (Kg/m^2^)	22.73	23.05
	(±3.72)	(±4.25)
	(N.V. 18.5–24.9)	(N.V. 18.5–24.9)
Waist circumference (cm)	83.83	74.85
	(±8.78)	(±7.19)
	(N.V. <94)	(N.V. <80)
Hip circumference (cm)	96.83	103.13
	(±6.25)	(±13.24)
waist-hip ratio (WHR)	0.86	0.72
	(±0.05)	(±0.05)
	(N.V. <1)	(N.V. <0.85)

*The table shows the values obtained from the standard anthropometric analysis. For each parameter, the mean values ± SD are presented. When possible, normal values (N.V.) were reported (M, men and W, women)*.

### Body composition analysis

Body composition analysis showed quantitative changes in all the partitions of the body. All the patients had a loss of muscle mass, as shown by a marked reduction of FFM, FFMI, and ALMI, and augmented FM, as indicated by % FM and FMI.

In particular, all patients were sarcopenic, based on ALMI, and seven were sarcopenic-obese, based on ALMI and % FM (Tables 2 and 3).

**Table 2:** **Dual-energy X-ray absorptiometry**.

	M (*n* = 3)	W (*n* = 5)
Fat-free mass (FFM) (%)	62.03	48.36
	(±18.19)	(±6.55)
Fat-free mass (FFM) (Kg)	42.39	29.22
	(±4.08)	(±3.53)
FFMI (FFM/height^2^) (Kg/m^2^)	14.43	10.90
	(±1.27)	(±0.85)
Fat mass (FM) (%)	34.63	51.64
	(±14.21)	(±6.55)
Fat mass (FM) (Kg)	23.86	31.93
	(±12.05)	(±9.76)
FMI (FM/height^2^) (Kg/m^2^)	8.18	12.07
	(±4.20)	(±3.71)
Bone mineral content (BMC) (%)	3.16	3.06
	(±0.61)	(±0.84)
Bone mineral content (BMC) (Kg)	2.08	1.82
	(±0.04)	(±0.36)
*T*-score	−1.87	−0.23
	(±0.49)	(±0.86)
Trunk-to-limb fat mass ratio	1.10	0.82
	(±0.03)	(±0.06)
ALMI (Kg/m^2^)	5.68	4.25
	(±0.57)	(±1.43)
ALM (Kg)	9.79	6.92
	(±1.01)	(±2.35)
Trunk lean mass (Kg)	21.35	14.66
	(±2.24)	(±1.12)
Trunk fat mass (Kg)	11.85	13.70
	(±6.13)	(±5.11)

*The table shows DXA body composition values. Mean values ± SD are presented (M, men and W, Women)*.

**Table 3:** **Body composition values obtained by anthropometric and DXA analyses, in BM and UCMD patients, compared to a healthy population**.

ID	Sex	Age (years)	BMI (Kg/m^2^)	Fat %	FFM%	BFMI (Kg/m^2^)	Trunk to limbs FM ratio	ALMI (Kg/m^2^)
Pt. 1	W	48	20.9	49.90 ↑	50.1 ↓	10.10 ↓	0.88 ↓	3.60 ↓
			N.V. (18.5–24.9)	N.V. (39.8–40.8)	N.V. (59.2–60.2)	N.V. (10.72–11.20)	N.V. (0.92–0.947)	N.V. (6.93–6.9)
Pt. 2	W	19	22.10	53.50 ↑	46.5 ↓	11.80 ↑	0.83 ↑	3.48 ↓
			N.V. (18.5–24.9)	N.V. (35.1)	N.V. (64.8)	N.V. (8.48)	N.V. (0.745)	N.V. (6.81)
Pt. 3	W	42	18.19	41.30 ↑	58.7 ↓	7.53 ↓	0.80 ↓	3.81 ↓
			N.V. (18.5–24.9)	N.V. (38.9–39.8)	N.V. (60.2–62.1)	N.V. (10.27–10.72)	N.V. (0.897–0.920)	N.V. (6.95–6.93)
Pt. 4	W	29	24.60	55.00 ↑	45 ↓	13.50 ↑	0.86 ↑	4.94 ↓
			N.V. (18.5–24.9)	N.V. (36–37)	N.V. (63–64)	N.V. (8.9–9.35)	N.V. (0.796–0.841)	N.V. (6.86–6.9)
Pt. 5	W	22	29.44	58.50 ↑	41.5 ↓	17.40 ↑	0.73 ↓	4.93 ↓
			N.V. (18.5–24.9)	N.V. (35.1–36)	N.V. (64–64.9)	N.V. (8.48–8.9)	N.V. (0.745–0.796)	N.V. (6.81–6.86)
Pt. 6	M	36	23.29	44.20 ↑	45.8 ↓	10.30 ↑	1.14 ↑	5.05 ↓
			N.V. (18.5–24.9)	N.V. (26.6–27.5)	N.V. (72.5–73.4)	N.V. (7.19–7.57)	N.V. (1.125–1.183)	N.V. (9.09–9.12)
Pt. 7	M	27	18.76	18.30 ↓	81.7 ↑	3.34 ↓	1.15 ↑	6.35 ↓
			N.V. (18.5–24.9)	N.V. (24.60–25.70)	N.V. (74.3–75.4)	N.V. (6.37–6.78)	N.V. (0.995–1.063)	N.V. (8.94–9.02)
Pt. 8	M	28	26.13	41.40 ↑	58.6 ↓	10.90 ↑	1.08 ↑	5.81 ↓
			N.V. (18.5–24.9)	N.V. (24.60–25.70)	N.V. (74.3–75.4)	N.V. (6.37–6.78)	N.V. (0.995–1.063)	N.V. (8.94–9.02)

*The table shows the body composition values of our subjects (M, men and F, women) compared to healthy values reported in the literature (Kelly et al., 2009; Wang et al., 2010). We used: “ ↑” when the values were increased; “ ↓”when the values were low*.

Moreover, from the DXA data (trunk-to-limb FM ratio) and anthropometric parameters (waist circumference and waist/hip ratio index), we can deduce that, in the two compartments, the increased fat tissue was equally distributed (DXA) compensating for the loss of muscle mass (circumferences).

Bone mineral content, the other component of the FFM, is also strongly reduced in these patients, especially in men. The data are confirmed by *T*-score calculations in our male patients (Male *T*-score −1.87). *T*-score is a diagnostic for osteopenia (Kanis et al., 2013) (Tables 2 and 3).

### Blood, urine biochemical analysis, and nitrogen balance

Blood and urine analysis do not show any specific pathological modification. In particular, although seven out of eight patients were obese, according to % fat determined by DXA, none had high levels of blood triglycerides, total cholesterol, HDL, or LDL (Jukema and Simoons, 1999). Blood creatinine levels were moderately decreased (Ballesteros et al., 1994), particularly in women. Urinary creatine levels were generally low, particularly in men. Consequently, calculated CHI, an index of muscle mass (Rosenfalck et al., 1994), was greatly reduced in UCMD and BM patients.

Nitrogen balance was in the normal range.

Moreover, megascore values in our patients reflect the low strength condition typical of MDs (Table 4).

**Table 4:** **Clinical and laboratory data**.

	Men (*n* = 3)	Women (*n* = 5)
Creatinine (mg/dL)	0.64	0.26
	(±0.30)	(±0.07)
	N.V. (0.8–1.3)	N.V. (0.6–1.1)
Urinary creatinine (mg/24 h)	893.33	394.00
	(±347.04)	(±107.84)
	N.V. (500–2000)	N.V. (500–2000)
Creatinine height index (CHI) (%)	57.63	39.19
	(±22.45)	(±10.83)
	N.V. (109)	N.V. (110)
Dietary CHO intake (g)	342.56	197.62
	(±48.17)	(±62.38)
Dietary lipid intake (g)	89.05	63.62
	(±27.47)0	(±27.55)
Dietary protein intake (g)	83.58	68.12
	(±8.90)	(±26.02)
Nitrogen intake (g)	13.37	10.90
	(±1.42)	(±4.16)
Urinary urea nitrogen (g)	13.04	11.99
	(±1.08)	(±3.48)
Nitrogen balance	−0.67	−1.84
	(±1.67)	(±1.30)
	N.V. (0.00)	N.V. (0.00)
Megascore	1093.33	572.60
	(±306.37)	(±233.11)

*The table shows the biochemical values obtained from blood and urine analysis and Megascore (Merlini et al., 2002, 2004). For each parameter, the mean values ±SD are presented. When possible, normal values (N.V.) were reported (M, men and W, women)*.

### Resting energy expenditure and respiratory quotient

**Figure 1:**
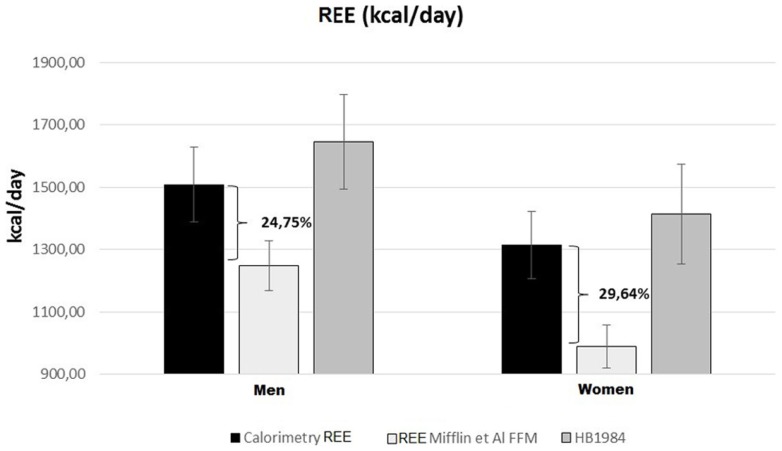
**Predicted and measured REE in UCMD and BM patients grouped by sex**. Mean REE measured with indirect calorimetry (black rectangles); REE estimated with FFM-based Mifflin equation (Mifflin et al., 1990) (white rectangles) or Harris-Benedict) revised equation (Roza and Shizgal, 1984) (gray rectangles).

Resting energy expenditure was measured by indirect calorimetry and estimated by specific predictive equations. The REEs estimated by the equations based on weight, height, age, and sex accurately predicted the same values measured with indirect calorimetry [Harris-Benedict and Schofield (“Energy and protein requirements. Report of a joint FAO/WHO/UNU Expert Consultation,” 1985; Roza and Shizgal, 1984)]. REE was instead severely underestimated using the FFM-based predictive equations of Mifflin and Katch and McArdle (McArdle et al., 1986; Mifflin et al., 1990). Hence, considering that these patients are characterized by a reduced FFM in kilograms, we can deduce that there is a relative hypermetabolic state (Müller, 2007) with a higher ratio of REE (kcal)/FFM (kg) (Figure 1).

**Figure 2:**
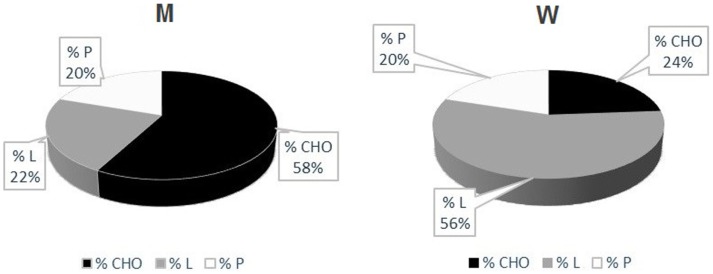
**UCMD and BM patients’ metabolism**. Percentage of Carbohydrates (CHO), Lipids (L) and Proteins (P) utilized during indirect calorimetry examination. These values were calculated from respiratory quotients and urine nitrogen measurement (Livesey and Elia, 1988) Mean values in men (left) and women (right) are reported.

Measured RQs values are indicative of a mixed nutrient-based metabolism. However, there was a sex difference concerning the type of substrates utilized. While male patients had a higher carbohydrate-based metabolism (57.67 ± 14.57% carbohydrates), women showed a higher lipid-based metabolism (56.00 ± 16.39% lipids) (Table 5) (Figure 2). There was a strong positive correlation between the quantity of FFM in kg and the percentage of carbohydrates metabolized during REE. This correlation, however, was negative for the percentage of lipids metabolized. No correlation was found between the percentage of proteins used as a metabolic substrate and FFM.

**Table 5:** **Energy balance**.

	M (*n* = 3)	W (*n* = 5)
REE indirect calorimetry (Kcal)	1508.33	1315.02
	(±120.03)	(±107.55)
REE Mifflin et Al (Kcal)	1248.09	988.67
	(±80.28)	(±69.62)
REE Katch and McArdle (Kcal)	1285.63	1001.19
	(±88.02)	(±76.34)
REE FAO Schofield et al. (Kcal)	1708.48	1392.91
	(±175.28)	(±173.51)
REE Harris-Benedict 1984 (Kcal)	1645.34	1412.83
	(±152.67)	(±159.62)
Mifflin FFM/REE	76.25	70.36
Indirect calorimetry %	(±8.28)	(±5.20)
FAO/REE indirect calorimetry %	113.15	106.35
	(±4.14)	(±15.14)
Harris-Benedict 1984/REE	109.01	107.88
Indirect calorimetry %	(±1.71)	(±14.32)
Respiratory quotient (RQ)	0.90	0.80
	(±0.06)	(±0.03)
RQ proteins (%)	19.67	20.00
	(±2.89)	(±9.89)
RQ lipids (%)	22.33	56.00
	(±17.95)	(±16.39)
RQ carbohydrates (%)	57.67	23.75
	(±14.57)	(±7.45)
REE/FFM (Kcal/Kg)	35.77	45.46
	(±3.92)	(±6.13)
REE/FM (Kcal/Kg)	80.61	44.54
	(±51.94)	(±13.75)

*In this table, values of indirect calorimetry and predictive equations analysis are presented. For each parameter, the mean values ± SD are presented (M, men and W, women)*.

**Figure 3:**
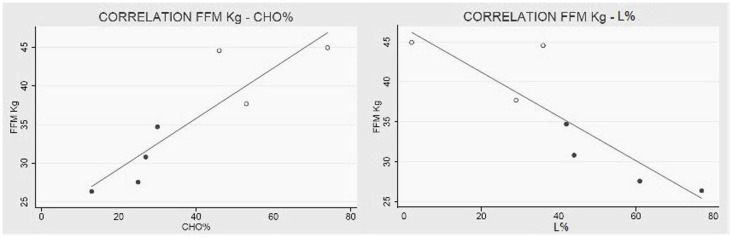
**Collagen VI myopathies: FFM metabolism**. (Left) Linear correlation between FFM in kg and percentage of carbohydrates used as metabolic substrate (correlation coefficient *R* = 0.89 e *P* < 0.01), and (right) correlation between FFM and percentage of lipids used as metabolic substrate (correlation coefficient *R* = −0.87 e *P* = 0.01). White dots represent men and black dots represent women. The percentage of substrate utilized was calculated using the respiratory quotient and urinary urea nitrogen (Livesey and Elia, 1988). Data from one BM were missing.

All in all, patients are characterized by an augmented REE per kilogram of FFM; additionally, subjects with higher FFM values metabolize more carbohydrates and less lipids then the ones with minor FFM levels (Figure 3).

### Muscle strength

Megascores expressed as the sum of the muscle strength of eight different tests, were 1093.33 (±306.37) Newton in men and 572.60 (±233.11) in women. These values were markedly reduced, as muscle strength was low in all muscle groups, compared with the normative values (Van der Ploeg et al., 1991; Beenakker et al., 2001).

**Figure 4:**
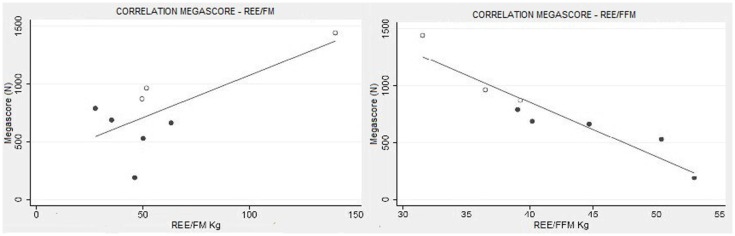
**Muscle strength and functional body composition**. As expected there was no correlation between Megascore and REE/FM, but instead a strong correlation between Megascore and the REE/FFM ratio (*R* = −0.94, *P* < 0.001) (right).

There was a strong correlation between muscle strength, expressed as Megascore, and the various indices of muscle mass, but no correlation with the indices of body fat. A strong correlation was found between Megascore and blood creatinine. This correlation was even stronger with urinary creatinine and the derived index “CHI” (Figure 6). UCMD and BM patients who had a higher FFM showed a better performance in the muscle strength tests. The Megascore was directly proportional to FFM in kilograms and inversely proportional to the REE/FFM ratio (Figure 4).

**Figure 5:**
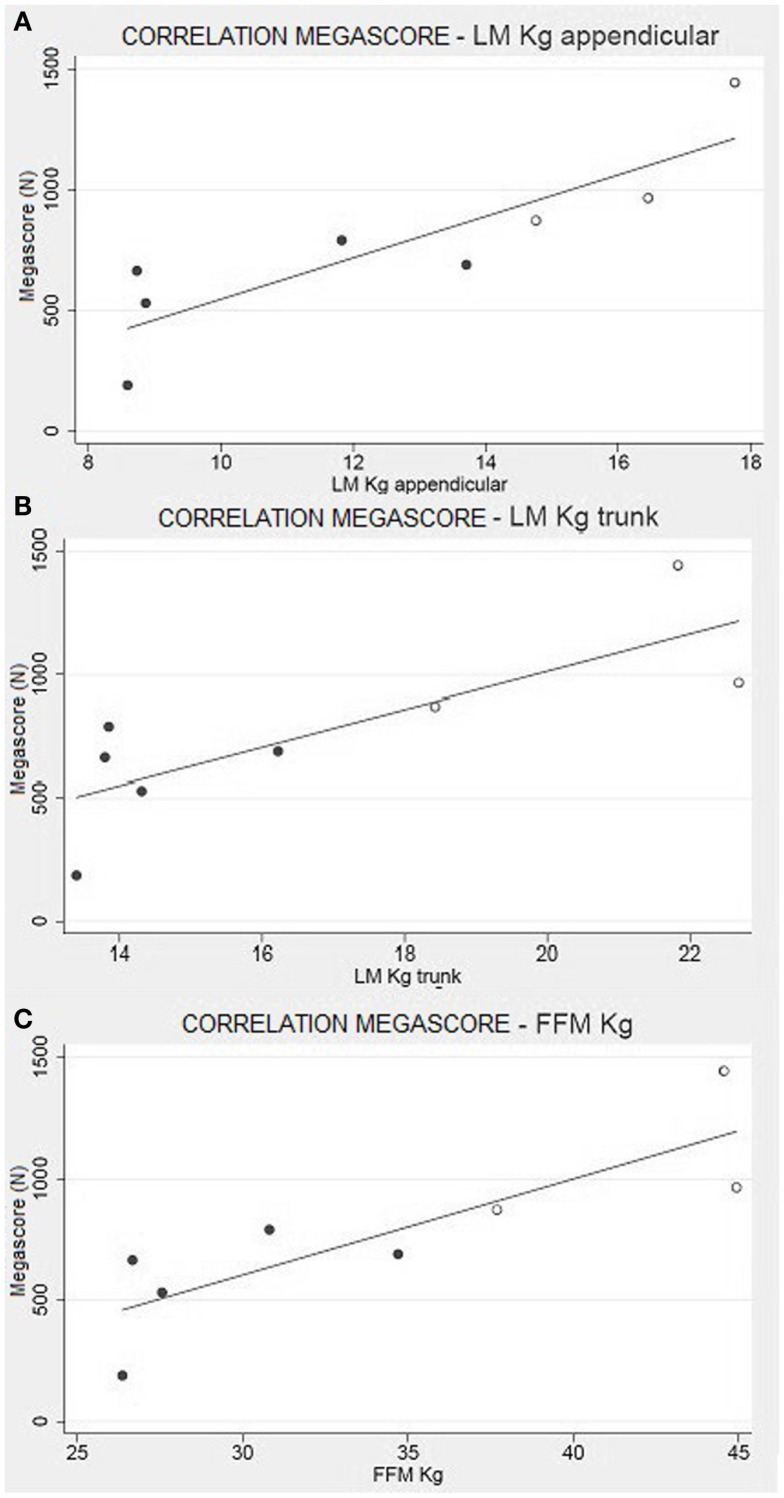
**Muscle strength and body composition**. Correlation between appendicular LM **(A)** and Trunk LM **(B)** with the Megascore [respectively for limbs and trunk, *R* = 0.87 (*P* < 0.01) and *R* = 0.80 (*P* < 0.05)]. **(C)** Correlation between total FFM in kg and Megascore (*R* = 0.84, *P* < 0.01).

This trend is maintained in the correlation between the Megascore and both trunk and limbs FFM. However, the linear correlation coefficient was higher between Megascore and appendicular FFM than Megascore and Trunk FFM (Figure 5). FFM% also correlated with Megascore, but the correlation was weaker.

No correlation was found any between the Megascore and the indices of fat mas, FM in kilograms, FM percentage, or REE/FM ratio.

## Discussion

In this study, we have investigated the relationship between body composition, energetic metabolism, and muscle strength in a cohort of patients with COL6-RM.

According to body composition, evaluated by DXA, all patients could be defined as sarcopenic and all but one as sarcopenic-obese. The patients showed a marked increment of the amount of FM and a severe loss of FFM without important modifications in the BMI, which ranged from underweight to overweight, or in waist circumferences measures, which were within a normal range. This peculiar modification in body composition can be explained by the process of fatty infiltration of the muscle in MD (Tuffery-Giraud et al., 2004; Jarraya et al., 2012; Willis et al., 2014).

Since FFM is the major determinant of energy expenditure in normal subjects, we integrated body composition analysis with the analysis of energy status and of muscle strength in accordance with the new concept of functional body composition (Müller et al., 2009).

COL6-RM patients showed a REE, analyzed by indirect calorimetry, in the range of normality, despite the severe reduction of the FFM. Predictive formulas estimate REE values in line with what we actually have found by indirect calorimetry. Applying the concept of functional body composition and relating FFM to energy expenditure, we found a clear deviation from normality (metabolic disequilibrium) with a considerably augmented REE per kilogram of FFM. Our assumptions are supported by the fact that REE, estimated through FFM-based equations, clearly underestimate the effective metabolism of these patients by 25–30% (Figure 1).

If we compare the metabolism predicted by FFM-based-formulas with the values of REE, adjusted for body composition, we find that the FFM of these patients is overworking or in a hypermetabolic state (Müller, 2007). A similar metabolic alteration has been reported by Zanardi et al. in Duchenne patients (Zanardi et al., 2003b). It has been considered an enduring enigma: why is the ratio of REE to metabolically active tissue mass, expressed as the REE/FFM ratio, greater in magnitude in subjects with a small FFM than in subjects with a large FFM? (Heymsfield et al., 2002).

In COL6-RM patients, FFM requires more energy than in healthy subjects, and because FFM is made up of muscle and internal organs, we can only speculate as to which of these two components presents a hypermetabolic state. In particular, the augmented REE/FFM ratio could be due to a non-physiological increase in visceral organ metabolism, or to an altered energy expenditure of muscle cells. However, even if our study cannot completely answer this question, all evidence suggests that the second hypothesis is the right one.

If we consider the pathological alteration of muscle structures, underlined in mouse model studies (De Palma et al., 2013), with the loss of muscular protein stability leads to a loss of efficiency, and additional energy is necessary to maintain muscle cell homeostasis. Even if this theory has been demonstrated in other studies no one had ever pointed out that this behavior is strictly correlated with the severity of the disease. Our hypothesis is that, in a dystrophic environment, the energy that the muscle has to spend in order to maintain its function is inversely proportional to the total muscular mass. Moreover, if the augmented REE/FFM were due to an increased metabolism of the visceral organs, we should have found some alterations in bio-humoral parameters (Gelfand et al., 1987; Izamis et al., 2012). On the contrary COL6-RM subjects do not show any important variation in blood or urine biochemical assays.

Additionally, there was no correlation between the REE/FM and the clinical status portrayed by Megascore analysis (Figure 4). Therefore, we can exclude increased adipocytes energy expenditure as a source of the hypermetabolic state.

Finally, recent discoveries about mitochondrial metabolism (Bernardi and Bonaldo, 2008; Shaham et al., 2010) offer a possible biochemical explanation of the increased REE/FFM ratio. In particular, Bernardi et al. have found important alterations in mitochondrial membrane permeability due to the ColA6 mutation. The lack of Collagen VI causes increased transient openings of the Permeability Transition Pore ion channel in the mitochondrial inner membrane, with consequent mitochondrial depolarization and energy dissipation. This leads to a switch of the ATP synthase into an ATP hydrolase with a progressive impairment of respiration which may be responsible for the augmented REE spent by the muscular mass. This pathophysiology mechanism may explain our findings.

We also discovered that the metabolic substrates consumed by these patients are strictly related to their FFM (Figures 2 and 3). In particular, patients with higher FFM have a carbohydrate-based metabolism, while the ones with lower FFM prevalently use fatty acids as metabolic substrates. These findings suggest that patients with a relatively greater FFM have more muscular mass, and consequently an increased glycogen reserve to be used, compared to patients with lower FFM (Tsujino et al., 2000). On the other hand, the fat-based metabolism is explained by the increasing fat infiltration with disease progression; the depletion of muscular mass and the correlated decrement in glycogen storage lead to a metabolic shift toward burning fatty acids, whose reserves increase and infiltrate muscular tissue (Tagliavini et al., 2014). These evidences suggest that the worsening of the pathology is closely correlated to important changes in muscle metabolism.

Another significant result is the correlation between FFM and muscle strength, summarized by the Megascore (Figure 5). Other studies have previously demonstrated that Megascore is a good indicator of patients’ muscular efficiency, paralleling muscle function (Merlini et al., 2002, 2004). Hence, the correlation between muscle Megascore and FFM is more a portrait of the muscular mass and its efficiency, rather than of visceral organ activity. The strong correlations between Megascore and appendicular LM, where the non-muscle component of FFM is minimal, support this hypothesis.

This is a perfect expression of the concept of functional body composition: in COL6-RM, a pure body composition parameter like appendicular LM is directly correlated to muscular strength (Megascore) and contributes to the diagnosis of sarcopenia, a condition in which the loss of skeletal muscle mass is associated with lower muscle strength and function (Abellan Baumgartner et al., 1998; van Kan et al., 2012)

**Figure 6:**
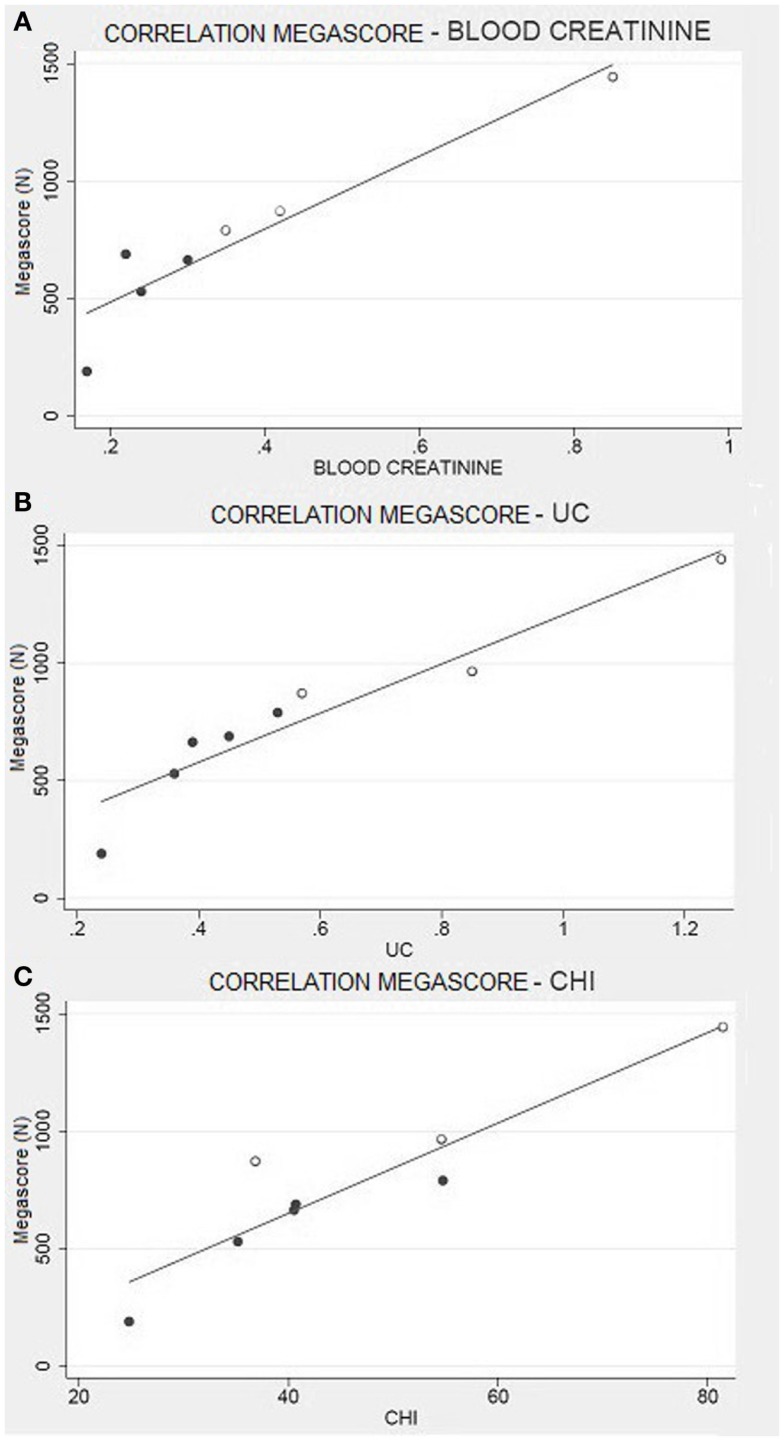
**Muscle strength and creatinine**. **(A)** Correlation between Megascore and blood creatinine (*R* = 0.94, *P* = 0.001), or **(B)** urine creatinine (UC) (*R* = 0.95, *P* < 0.001). **(C)** Correlation between Megascore and CHI (Rosenfalck et al., 1994) (*R* = 0.93, *P* = 0.001).

Creatinine is derived from the metabolism of creatine, which is transformed into phosphocreatine and used by muscles as an energetic substrate (Hoagland et al., 1945). A higher production of creatinine is linked to a higher use of phosphocreatine and, consequently, to greater muscular efficiency. In order to confirm this hypothesis, and to exclude any artifact due to individual body composition, we have analyzed the CHI, which evaluates urinary creatinine levels by normalizing the individual differences among subjects (Rosenfalck et al., 1994). Even in this case, the CHI scores perfectly correlate to the muscle strength, as evaluated by Megascore (Figure 6).

Hence, even if Franciotta et al. (2003) declare that urinary creatinine is not a good predictive indicator of skeletal muscular mass in Duchenne dystrophy; our results suggest that it is a good indicator of muscular performance.

Another important correlation is the one between blood creatinine and Megascore. Even if we have to consider renal filtration, blood creatinine gives an instant picture of the muscle metabolism (Baxmann et al., 2008). The probability of finding circulating creatinine is directly proportional to the quantity of phosphocreatine produced and used by muscle cells. Considering our results about urinary creatinine and CHI, this finding confirms the previous ones.

All in all, in this study, we have pointed out the importance of a nutritional approach to genetically based pathologies, such as UCMD and BM diseases. Additionally, we have underlined the necessity of a functional body composition analysis, which could be a powerful clinical tool for patients’ follow-up and prognosis.

The main limit of our study is represented by the scant number of recruited patients, caused by the rarity of these pathologies; hence, our conclusions should be confirmed by the analysis of a wider sample of subjects.

Our results confirm and complete what has been reported in the literature about collagen VI myopathies, further supporting the rationale for nutritional interventions aimed at correcting the metabolic imbalance and maintaining the patient’s muscular mass.

## Author Contributions

Silvia Toni made substantial contributions to acquisition of the data, carried out the nutritional evaluation, and performed the statistical analysis. Riccardo Morandi and Marcello Busacchi substantially contributed by discussing the data, writing the manuscript, and performing statistical analysis. Lucia Tardini contributed to the acquisition of data and carried out the anthropometric evaluation. Luciano Merlini conceived the study and participated in its design and coordination. Nino Carlo Battistini evaluated the body composition data. Massimo Pellegrini participated in the design of the study, contributed to the statistical analysis, and has contributed substantially in the interpretation of data.

## Conflict of Interest Statement

The authors declare that the research was conducted in the absence of any commercial or financial relationships that could be construed as a potential conflict of interest.
